# Supplementary material for: Perceived needs of disease vector control programs: A review and synthesis of (sub)national assessments from South Asia and the Middle East
Source: PLoS Negl Trop Dis. 2024 Apr 17;18(4):e0011451. doi: 10.1371/journal.pntd.0011451 (PMC11075900; doi:10.1371/journal.pntd.0011451)
Supplement: S1 Appendix — (DOCX) [file pntd.0011451.s001.docx]

**S1 Appendix. Panel of stakeholders involved in vector control needs assessment in each country/state.**

Bangladesh:

- Agriculture Extension Department, Ministry of Agriculture
- Bangladesh Council of Scientific and Industrial Research
- Bangladesh Crop protection Association
- Bangladesh Parjaton Corporation, Environment Department
- Bangladesh Standards and Testing Institution
- Chattogram City Corporation
- Chattogram Port Authority
- Chattogram University
- Dhaka North City Corporation
- Dhaka South City Corporation
- Dhaka University
- Directorate General of Health Services, Ministry of Health & Family Welfare
- Forest and Climate Change
- Gazipur City Corporation
- International Centre for Diarrhoeal Disease Research-Bangladesh
- Jahangirnagar University
- Malaria and Aedes Transmitted Diseases Control Programme, Directorate General of Health Services, Ministry of Health & Family Welfare
- Ministry of Civil Aviation and Tourism
- Ministry of Commerce
- Ministry of Environment
- Ministry of Industries
- Ministry of Local Government
- Moulvibazar Municipality
- Plant Protection Wing, Ministry of Agriculture
- Rural Development and Co-operatives
- Savar Municipality

Assam state (India):

- Indian Council of Medical Research-National Institute of Malaria Research, New Delhi
- National Institute of Malaria Research, Guwahati, Assam
- National Vector Borne Disease Control Programme, Assam
- Tea State managers of Kumarikata, district Baksa, and Balijon, district Dibrugarh
- Zonal Entomologists, National Vector Borne Disease Control Programme, Assam

Gujarat state (India):

- Ahmedabad Municipal Corporation
- Commissionerate of Health & Family Welfare, Gandhinagar
- District Malaria Officer, Gandhinagar
- ICMR-National Institute of Malaria Research, Field Station, Nadiad
- ICMR-National Institute of Malaria Research, New Delhi.
- Regional Entomologist, Vadodara
- Research Officer, Regional Office of Heath and Family Welfare, Ahmedabad
- State Entomologist, Gandhinagar
- State Representation, National Vector Borne Disease Control Programme
- Surat Municipal Corporation

Jharkhand state (India):

- Department of Health, Medical Education and Family Welfare, Jharkhand
- District Malaria/Vector Borne Disease Office, Hazaribag, Jharkhand
- State Programme Office, Directorate of National Vector Borne Disease Control Programme, Jharkhand

Tamil Nadu state (India):

- Department of Health Research, Ministry of Health and Family Welfare, Puducherry
- Directorate of Public Health and Preventive Medicine, Chennai
- Greater Chennai Corporation
- Indian Council of Medical Research, Vector Control Research Centre, Puducherry
- National Institute of Malaria Research, Field Station, Chennai
- Public Health and Preventive Medicine, Tamil Nadu
- State Programme Office, Directorate of National Vector Borne Disease Control Programme

Iran:

- Department of Environment
- Environmental and Occupational Health Centre
- Food and Drug Administration
- Ministry of Health and Medical Education, Centers for Disease Control
- National Veterinary Organization
- Pasteur Institute of Iran
- Plant Protection Organization
- School of Public Health, Tehran University of Medical Science

Iraq:

- Disease Vector Control Section, Public Health Department, Ministry of Health
- National Committee for Registration and Approval of Pesticides, Ministry of Agriculture
- Research and Development Department, Ministry of Higher Education and Scientific Research
- Technical Department, Ministry of Environment
- Technical Committee for Integrated Management for Disease Vector Control
- Research and Development Department, Ministry of Education
- Ministry of Construction, Housing, Municipalities and Public Works
- Baghdad Municipality
- Department of Planning and Follow-up, Ministry of Transportation
- Expert from WHO Regional Office
- An expert doctor

Maldives:

- Health Protection Agency
- Local Government Authority
- Ministry of Fisheries, Marine Resources and Agriculture
- National Health Laboratory within Maldives Food and Drug Authority
- Water and Environment Management Section, Health Protection Agency
- Environmental Protection Agency
- Maldives National University
- Ministry of Environment
- Maldives National Defence Force, Ministry of Defence
- Ministry of Defence
- Maldives Customs Service
- Male’ City Council

Nepal:

- ASCEND Nepal
- B.P. Koirala Institute of Health Sciences, Dharan
- Epidemiology and Disease Control Division, Department of Health Services
- FAIRMED Foundation, Nepal
- Global Fund Malaria Programme
- Health Division, Ministry of Social Development
- Health Office, Morang
- Health Office, Saptari
- Neglected Tropical Diseases & Vector-Borne Diseases Control Section
- Nepal Health Research Council
- Provincial Health Directorates
- Public Health and Infectious Disease Research Center, Kathmandu
- Vector-Borne Disease Research and Training Centre, Hetauda
- World Health Organization, Nepal Country Office

Sri Lanka:

- Agriculture Department
- Anti Filariasis Campaign
- Anti Malaria Campaign
- Colombo Municipal Council
- Disaster Unit, Ministry of Health
- Environmental Department
- Epidemiology Unit, Ministry of Health
- Ministry of Health
- National Dengue Control Unit
- Registrar of Pesticide Office
- University of Peradeniya
- University of Ragama

Yemen:

- Ministry of Public Health and Population
- National Malaria Control Programme
- National Schistosomiasis Control Programme
- Dengue and Chikungunya Unit based at National Malaria Control Programme
- Leishmaniasis program
- Plant Protection Department, Ministry of Agriculture and Irrigation
- Environment Protection Authority, Ministry of Water & Environment
- Country Office, World Health Organization, Sana’a
