# Supplementary material for: Perceived needs of disease vector control programs: A review and synthesis of (sub)national assessments from South Asia and the Middle East
Source: PLoS Negl Trop Dis. 2024 Apr 17;18(4):e0011451. doi: 10.1371/journal.pntd.0011451 (PMC11075900; doi:10.1371/journal.pntd.0011451)
Supplement: S2 Appendix — (DOCX) [file pntd.0011451.s002.docx]

**S2 Appendix. List of vector control needs assessment reports, by country, with availability from third party sources.**

Bangladesh:

Report: Report of the Vector Control Needs Assessment (VCNA) in Bangladesh, December 2020. By Kabirul Bashar and Rajib Chowdhury. Unpublished report, 96 pp.

Available from: World Health Organization, Country Office for Bangladesh, House-SW(I) 1/A, Road 8, Gulshan 1, Dhaka-1212, Bangladesh. E-mail: sebanregistry@who.int

India:

Reports:

Vector Control Needs Assessment (VCNA) under National Vector Borne Disease Control programme, Assam State, India, September 2020. By Hardev Prasad Gupta. Unpublished report, 30 pp.

Report on the Vector Control Needs Assessment (VCNA) in Gujarat State, India, September 2020. By Rajendra M. Bhatt. Unpublished report, 41 pp.

Jharkhand Vector Control Needs Assessment, September 2020. By A.T.S. Sinha. Unpublished report, 29 pp.

Vector Control Needs Assessment, Tamil Nadu, India, November 2020. By Ashwani Kumar, S. Sabesan, A.N. Sriram and K.H.K. Raju. Unpublished report, 56 pp.

Available from: WHO Representative’s Office, 537, A Wing, Nirman Bhawan, Maulana Azad Road, New Delhi 110 011, India. E-mail: wrindia@who.int

Iran:

Report: Vector Control Needs Assessment (VCNA), Islamic Republic of Iran, 2018. By Mohammad Mehdi Sedaghat and Ahmadali Enayati. Unpublished report, 152 pp.

Available from: WHO Country Office for Islamic Republic of Iran, Tehran, Iran. E-mail: emacoirawr@who.int

Iraq:

Report: Vector control needs assessment questionnaire – Iraq, 2018. By Abdul Jaleel Naji Alzubaidi. Unpublished report, 70 pp.

Available from: WHO Representative’s Office, WHO Country Office, UN Compound, Near Diwan School, International Green Zone, Baghdad, Iraq. E-mail: emwroirq@who.int.

Maldives:

Report: Vector Control Needs Assessment – Maldives, 2020. By Aishath Shaheen Najmee and Sana Saleem. Unpublished report, 16 pp.

Available from: WHO Representative Office, Roashanee Building, 6th Floor, Male 20184, Republic of Maldives. E-mail: sewhomav@who.int

Nepal:

Report: Vector Control Needs Assessment for Nepal, 2021. By: Anonymous. Unpublished report, 54 pp.

Available from: World Health Organization, Country Office for Nepal, United Nations House, Pulchowk, Lalitpur, Kathmandu, Nepal. E-mail: senepwr@who.int

Sri Lanka:

Report: National Vector Control Needs Assessment for Vector Borne Disease Control Programmes in Sri Lanka, September 2020. By P.H.D. Kusumawathie and Devika Perera. Unpublished report, 174 pp.

Available from: WHO Country Office for Sri Lanka, 5 Anderson Road, Colombo 05, Sri Lanka. E-mail: sesrlregistry@who.int

Yemen:

Report: Vector Control Needs Assessment: Yemen, June 2018. By Mohammed Abdurrahman. Unpublished report, 121 pp.

Available from: WHO Country Office for Yemen, 543 Sana’a, Yemen. E-mail: emacoyemwr@who.int
