# Supplementary material for: Perceived needs of disease vector control programs: A review and synthesis of (sub)national assessments from South Asia and the Middle East
Source: PLoS Negl Trop Dis. 2024 Apr 17;18(4):e0011451. doi: 10.1371/journal.pntd.0011451 (PMC11075900; doi:10.1371/journal.pntd.0011451)
Supplement: S1 Table — (DOCX) [file pntd.0011451.s003.docx]

**S1 Table. Description of the key items of the vector control system.**

| **Category** | **Key item** | **Description** |
| --- | --- | --- |
| **Impact & outcomes** | Disease impact | Impact of control operations on the incidence of disease over time |
|  | Vector outcomes | Outcome of operations on the occurrence or density of disease vectors over time |
| **Activities** |  |  |
| 1. *Scaling-up, integrating vector control* | Coverage | Proportion of coverage of the at-risk human populations with vector control interventions |
|  | Operational quality | Quality of implementation of vector control interventions |
|  | Pesticide management | Regulatory control, proper handling, supply, transport, storage, application and disposal of pesticide waste to minimize adverse environmental effects and human exposure |
| 1. *Vector surveillance and M&E* | Routine entomological surveys | Surveys of relevant entomological indicators (e.g. composition, density, seasonality, behavioural characteristics) at an appropriate frequency |
|  | Routine insecticide susceptibility tests | Standard bioassays on insecticide susceptibility of main disease vectors to insecticides available and/or used for vector control conducted at a regular frequency (e.g. annually) |
|  | Sentinel sites | Representative locations for monitoring of temporal changes in specific entomological indicators (e.g. insecticide susceptibility) |
|  | M&E of operations | Monitoring and evaluation of the quality and timeliness of implementation of vector control interventions |
|  | Data management and sharing | Management of vector control and surveillance data in appropriate data systems and sharing of data or results among relevant partners and stakeholders |
| 1. *Community mobilization* | Systematic implementation | Organized and methodical implementation of community mobilization activities |
|  | Monitoring | Monitoring of the level and quality of community mobilization activities |
| 1. *Intra/intersectoral collaboration* | Cross-disease collaboration | Collaboration on vector control or surveillance between disease-specific programs |
|  | Intersectoral committee | An existing and functional committee with representatives from relevant sectors, including sectors other than health, that is tasked with overseeing vector control and prevention |
|  | Intersectoral action | Activities on vector control and prevention conducted with involvement of sectors other than health |
| **Inputs** | Entomologists | Availability of personnel with appropriate expertise for public health entomology or vector biology |
|  | Vector control operators | Availability of personnel trained on implementation of vector control interventions |
|  | Training, entomology | Availability of opportunities for training or education on public health entomology |
|  | Training, operations | Availability of training curricula and training events on implementation of vector control interventions |
|  | Entomology laboratories | Laboratory infrastructure to support entomological surveillance activities, including insecticide resistance monitoring |
|  | Research input | Research results that are adopted to benefit vector control or surveillance activities |
| **Enabling factors** | Strategic plans | Plans that describe how a disease-control strategy should be implemented to achieve the set targets |
|  | Guidelines | National guidelines that describe how vector control or surveillance activities should be implemented |
|  | Organizational structure | Structure for organizing vector control and surveillance activities between institutional entities and administrative levels |
