# Supplementary material for: Perceived needs of disease vector control programs: A review and synthesis of (sub)national assessments from South Asia and the Middle East
Source: PLoS Negl Trop Dis. 2024 Apr 17;18(4):e0011451. doi: 10.1371/journal.pntd.0011451 (PMC11075900; doi:10.1371/journal.pntd.0011451)
Supplement: S1 Data — (DOCX) [file pntd.0011451.s004.docx]

**S1 Data. Table containing the underlying numerical data for Fig 2.**

|  | Malaria |  | Dengue |  | Leishmaniasis | |
| --- | --- | --- | --- | --- | --- | --- |
|  | Mean | SE | Mean | SE | Mean | SE |
| Impacts, outcomes | 0.88889 | 0.111111 | -1 | 0 | 0.6 | 0.4 |
| Scaling-up interventions | 0.35 | 0.194762 | 0.22917 | 0.201526 | 0.4 | 0.187083 |
| Vector surveillance and M&E | -0.06167 | 0.202455 | -0.19583 | 0.20123 | -0.28 | 0.338231 |
| Community mobilization | -0.15 | 0.183333 | -0.0625 | 0.257694 | -0.3 | 0.254951 |
| Intra/intersectoral collaboration | -0.05 | 0.216667 | 0.375 | 0.182981 | 0.1 | 0.276887 |
| Inputs | -0.02333 | 0.163303 | -0.10417 | 0.133398 | -0.0477 | 0.113333 |
| Enabling factors | 0.75 | 0.10319 | 0.6875 | 0.16195 | 0.8 | 0.08165 |
|  |  |  |  |  |  |  |
|  |  |  |  |  |  |  |
|  |  |  |  |  |  |  |
|  |  |  |  |  |  |  |
|  |  |  |  |  |  |  |
|  |  |  |  |  |  |  |

|  |  |  |  |  |  |  |
| --- | --- | --- | --- | --- | --- | --- |
