# Supplementary material for: Perceived needs of disease vector control programs: A review and synthesis of (sub)national assessments from South Asia and the Middle East
Source: PLoS Negl Trop Dis. 2024 Apr 17;18(4):e0011451. doi: 10.1371/journal.pntd.0011451 (PMC11075900; doi:10.1371/journal.pntd.0011451)
Supplement: Attachment — Submitted filename: Response letter_revised.docx [file pntd.0011451.s005.docx]

Response letter dated 17 March 2024

"Perceived needs of disease vector control programs: A review and synthesis of (sub)national assessments from South Asia and the Middle East"

PNTD-D-23-00718

**Below we provide our responses in bold font to the editor’s comments as well as to the reviewers’ comments.**

Email from Editor (E Janelli), 15 March 2024:

Is this paper more about the methodology/framework rather than the actual country data? Will the description of the analytical framework allow interested readers to replicate their study with other countries? If so, please do provide a data availability statement.

**Response: We confirm that the paper is primarily about testing the methodology and framework of the vector control needs assessment. The actual country data are presented to demonstrate what the use of the framework can deliver so that other countries can replicate the methods. Clearly, the data cannot be replicated by other countries because of different contextual conditions. Accordingly, we have provided the following data availability statement in the revised manuscript.**

**“Data availability: The data contained in the reports on which this work was based cannot be shared because they are property of third parties and the informed consent did not include the permission. The reports can be requested from the third-party sources listed in the S2 Appendix by researchers who meet the criteria for access to confidential data.”**

Email from Ms Anitha Samidurai’s dated 13 February 2024:

Please ensure that the CRediT author contributions listed for every co-author are completed accurately and in full.

**Response: Done**

Please ensure that the funders and grant numbers match between the Financial Disclosure field and the Funding Information tab in your submission form. Note that the funders must be provided in the same order in both places as well. Currently, " The WHO Global Malaria Programme" is missing from the Funding Information.

**Response: done**

Data availability

**Response: see above in response to the email from the Editor (E Janelli), 15 March 2024**

We do not publish any copyright or trademark symbols that usually accompany proprietary names, eg (R), (C), or TM (e.g. next to drug or reagent names). Please remove all instances of trademark/copyright symbols throughout the text, including 
© on line no 581.

**Response: done**

Our previously submitted responses to reviewers’ comments

*Reviewer's Responses to Questions:*

Key Review Criteria Required for Acceptance?
As you describe the new analyses required for acceptance, please consider the following:

Methods
-Are the objectives of the study clearly articulated with a clear testable hypothesis stated?

-Is the study design appropriate to address the stated objectives?
-Is the population clearly described and appropriate for the hypothesis being tested?
-Is the sample size sufficient to ensure adequate power to address the hypothesis being tested?
-Were correct statistical analysis used to support conclusions?
-Are there concerns about ethical or regulatory requirements being met?

Reviewer #1: (No Response)

--------------------
Results
-Does the analysis presented match the analysis plan?
-Are the results clearly and completely presented?
-Are the figures (Tables, Images) of sufficient quality for clarity?

Reviewer #1: (No Response)

--------------------
Conclusions
-Are the conclusions supported by the data presented?
-Are the limitations of analysis clearly described?
-Do the authors discuss how these data can be helpful to advance our understanding of the topic under study?
-Is public health relevance addressed?

Reviewer #1: (No Response)

Reviewer #2: Yes though I would like to see more input from the authors based on their experience and their knowledge of the resources needed. without it, the value of the info collected is undermined to some extent.

**Response: The reviewer is referring to the resources needed to fill gaps in vector control inputs and activities, which will depend strongly on the country context. We added to the Discussion: “****There has been a shortage of international resources to support national vector control systems [27]. Substantial investment will be needed in most of the countries/states to strengthen their capacity for vector surveillance and insecticide resistance monitoring using domestic and international funding sources.” (line 494). Reference [27] was added.**

--------------------
Editorial and Data Presentation Modifications?
Use  this section for editorial suggestions as well as relatively minor modifications of existing data that would enhance clarity. If the only modifications needed are minor and/or editorial, you may wish to recommend “Minor Revision” or “Accept”.

Reviewer #1: (No Response)

Reviewer #2: N/A

--------------------
Summary and General Comments
Use  this section to provide overall comments, discuss strengths/weaknesses of the study, novelty, significance, general execution and scholarship. You may also include additional comments for the author, including concerns about dual publication, research ethics, or publication ethics. If requesting major revision, please articulate the new experiments that are needed.

Reviewer #1: The manuscript explores the perceived needs of disease vector control programs in the South Asia and Middle East zones. Addressing vector control issues is crucial, particularly in regions where vector-borne diseases (VBD) are not yet under control. For those regions where control is established, more surveillance is essential to prevent resurgence. However, the presentation of the paper could be adjusted to better emphasize its contribution in transforming the pooling of country reports on the assessment of vector control needs into a research manuscript.

To enhance the manuscript's value, consider dividing it into two parts. The first part could focus on reviewing the state of the art regarding vector control in the countries covered.

**Response: We renamed the first section under Results: “Vector control state of the art” (line 274). We created a second section under Results: “(Sub)national assessments’ (line 311). The sections “Impacts and outcomes”, “Activities”, “Inputs”, and “Enabling factors” became subsections under “National assessments”. The third section under Results is “Synthesis” (unchanged).**

Important literature, including information about vector control history, current status, insecticides used, application periods, and resistance to insecticides, may provide a more comprehensive context.

**Response: In the section “Vector control state of the art”, we have considered the reviewer’s suggestion to expand information about aspects of vector control, however, we opted to keep the paper concise and focused on the needs assessment. We address each item separately:**

**Vector control history: This subject was not part of the vector control needs assessment reports. A literature review on the historical perspective for each country/state was beyond the scope of our study which focused on the current needs.**

**Current status: This is the subject of the national vector control assessments. This now constitutes section two under the Results.**

**Insecticide use: This is a complex topic, and we have recently provided a detailed account of global vector control insecticide use in a separate study (**[**https://doi.org/10.1038/s41598-021-03367-9**](https://doi.org/10.1038/s41598-021-03367-9) **), although WHO does not publicize country-specific insecticide use data. To address this topic of insecticide use, we added: ”Data on the use of vector control insecticide products were mostly absent from the assessment reports but a recent WHO study found that vector control insecticide use in the Asia-Pacific Region consisted mostly of pyrethroids and organochlorines for the control of malaria and leishmaniasis, and pyrethroids and organophosphates for the control of dengue [26].“ (line 282). Reference [26] was added.**

**Application periods: this is another complex topic, that would require its own study, if data were collected or available.**

**Insecticide resistance: This is another complex topic that would require a review for each country/state that differentiates variables such as vector species, insecticide class, testing methods, locations/areas, time of testing. This would be well beyond the scope of our study. We added the text: “Several countries/states expressed concern about the development of insecticide resistance in disease vectors of malaria, dengue and leishmaniases, but data on insecticide susceptibility testing were not systematically reported.” (line 285).**

The second part could present the synthesis of the national assessment per country, as it is currently structured. Countries vary in terms of vector control implementation, with only a few having programs for at least one of the three diseases. Table 1 could be more comprehensive by including the number of deaths per country and per disease.

**Response: In Table 1, we inserted a new column for disease deaths. We added the necessary details in the methods (line 234).**

Additionally, Graph 2 needs improvement in terms of clarity and legibility. Consider using either colors or letters consistently and ensure that the legend is not redundant.

**Response: This comment refers to Table 2, not Fig. 2. The comment applies equally to Table 3 and 4. In response, we improved the clarity of Tables 2–4 by using colours only, instead of colours plus letters (a,b,c). This removes redundancy. The footnotes of the tables were adjusted as needed.**

Most of the terminologies and concepts used need clarification (Table 2-4).

**Response: We added a new S1 Table “Description of the key items of the vector control system.” This describes each key item. In the Methods, we added: “(see descriptions in the S1 Table)” (line 247). Also, in tables 2–4, we added a footnote: “Key items are described in the S1 Table.”**

The quantitative analysis of perception appears more qualitative, and the significant disparities between these countries raise questions about the added value of pooling data from different countries together.

**Response: In the Methods, we changed the phrase “qualitative analysis” and instead wrote “A pooled analysis of the perceptions across countries/states…” (line 258). To address the reviewer’s concern, we added a sentence: “The purpose of this analysis was to study the average and variability of results in the set of (sub)national assessments, while noting that substantial differences in size, population and vector control conditions exist between countries/states.” (line 261).**

While acknowledging the limitations of the study is appropriate, consider expanding on them to guide readers on parameters that should be approached with caution, particularly in the synthesis.

**Response: We added: “A limitation in the synthesis of results was the disparities in size, population and vector control conditions between countries/states.” (line 572).**

Reviewer #2: Viewing the scale involved, the size of the populations of the nations included, the involvement of WHO plus the magnitude/ importance and value of the NTD Information reported, the article would be unusual, but highly appropriate when published in PLOS NTDs.

**Response: We agree with this observation; no change needed.**

--------------------
Figure Files:
While revising your submission, please upload your figure files to the Preflight Analysis and Conversion Engine (PACE) digital diagnostic tool, [https://pacev2.apexcovantage.com](https://pacev2.apexcovantage.com/). PACE helps ensure that figures meet PLOS requirements. To use PACE, you must first register as a user. Then, login and navigate to the UPLOAD tab, where you will find detailed instructions on how to use the tool. If you encounter any issues or have any questions when using PACE, please email us at [figures@plos.org](mailto:figures@plos.org).

**Response: We have uploaded Fig. 1 and 2 to the PACE, and we are attaching the figure files as downloaded through this tool.**

Data Requirements:
Please note that, as a condition of publication, PLOS' data policy requires that you make available all data used to draw the conclusions outlined in your manuscript. Data must be deposited in an appropriate repository, included within the body of the manuscript, or uploaded as supporting information. This includes all numerical values that were used to generate graphs, histograms etc.. For an example see here: <http://www.plosbiology.org/article/info%3Adoi%2F10.1371%2Fjournal.pbio.1001908#s5>.

**Response: At the end of the Methods, we added: “The numerical data used in Fig. 2 are included in S1 Data.” (line 266). In the Supplementary File, we added S1 Data.**

Reproducibility:
To enhance the reproducibility of your results, we recommend that you deposit your laboratory protocols in [protocols.io](http://protocols.io/), where a protocol can be assigned its own identifier (DOI) such that it can be cited independently in the future. Additionally, PLOS ONE offers an option to publish peer-reviewed clinical study protocols. Read more information on sharing protocols at <https://plos.org/protocols?utm_medium=editorial-email&utm_source=authorletters&utm_campaign=protocols>

**Response: Not applicable.**

References
Please review your reference list to ensure that it is complete and correct. If you have cited papers that have been retracted, please include the rationale for doing so in the manuscript text, or remove these references and replace them with relevant current references. Any changes to the reference list should be mentioned in the rebuttal letter that accompanies your revised manuscript. If you need to cite a retracted article, indicate the article's retracted status in the References list and also include a citation and full reference for the retraction notice.

**Response: References [26] and [27] were added in accordance with our responses to the reviewer comments. DOI and links were added to references.**

We look forward to receiving your decision.

Yours sincerely,

Rajpal S. Yadav

Department of Control of Neglected Tropical Diseases

World Health Organization, Geneva, Switzerland
